# The Expression of Uncoupling Protein 3 Coincides With the Fatty Acid Oxidation Type of Metabolism in Adult Murine Heart

**DOI:** 10.3389/fphys.2018.00747

**Published:** 2018-06-22

**Authors:** Karolina E. Hilse, Anne Rupprecht, Monika Egerbacher, Sarah Bardakji, Lars Zimmermann, Andrea E. M. Seiler Wulczyn, Elena E. Pohl

**Affiliations:** ^1^Department of Biomedical Sciences, Institute of Physiology, Pathophysiology and Biophysics, University of Veterinary Medicine Vienna, Vienna, Austria; ^2^Histology and Embryology, Department of Pathobiology, University of Veterinary Medicine Vienna, Vienna, Austria; ^3^German Centre for the Protection of Laboratory Animals (Bf3R), Federal Institute for Risk Assessment (BfR), Berlin, Germany

**Keywords:** respiratory chain proteins, oxygen consumption rate (OCR), mitochondrial morphology, lipid droplets, cell metabolism, mESC-derived cardiomyocytes

## Abstract

The involvement of mitochondrial uncoupling proteins 2 and 3 in the pathogenesis of cardiovascular diseases is widely acknowledged. However, contradictory reports show that the functions of UCP2/UCP3 are still disputed. We have previously described that UCP2 is highly abundant in cells that rely on glycolysis, such as stem, cancer and activated immune cells. In contrast, high amounts of UCP3 are present in brown adipose tissue, followed by heart and skeletal muscles - all known to metabolize fatty acids (FA) to a high extent. Using two different models – mouse embryonic stem cell (mESC) differentiation to cardiomyocytes (CM) and murine heart at different developmental stages – we now tested the concept that the expression ratio between UCP2 and UCP3 indicates the metabolism type in CM. Our results revealed the tight correlation between UCP3 abundance, expression of mitochondrial fatty acid oxidation (FAO) markers and presence of multiple connections between mitochondria and lipid droplets. We further demonstrated that the time course of UCP3 expression neither coincided with the onset of the electrical activity in CM, derived from mESC, nor with the expression of respiratory chain proteins, the observation which rendered protein participation in ROS regulation unlikely. The present data imply that UCP3 may facilitate FAO by transporting FAs into mitochondria. In contrast, UCP2 was highly abundant at early stages of heart development and in mESC. Understanding, that the expression patterns of UCP3 and UCP2 in heart during development reflect the type of the cell metabolism is key to the uncovering their different functions. Their expression ratio may be an important diagnostic criterion for the degree of CM differentiation and/or severity of a heart failure.

## Introduction

One of cardiovascular disease’s important hallmarks is mitochondrial dysfunction resulting in abnormal energy metabolism and increased ROS production in CM (Madamanchi and Runge, 2007). Members of the mitochondrial uncoupling protein family, UCP2 and UCP3, are thought to be beneficial in heart failure mainly by reducing ROS through mild uncoupling. However, despite extensive research in many laboratories, the function and even abundance of these two highly homologous proteins at different stages of heart development and in heart diseases remain elusive. Several studies failed to describe a clear phenotype for both genetic models UCP2^-/-^ and UCP3^-/-^, mainly because they generally do not take into account that diseases (especially metabolic) may have polygenic origins and/or be affected by environmental factors (Harper and Himms-Hagen, 2001). It was reported that a decrease or loss of UCP3 in rodent knockout models diminished the contractile heart function, increased ROS production and tissue damage following ischemia (Harmancey et al., 2013; Ozcan et al., 2013; Perrino et al., 2013), whereas other research groups demonstrated that a lack of UCP3 led to a higher death rate in mice due to cardiac arrhythmias upon lipid-challenged conditions (Nabben et al., 2014).

It is now becoming obvious that (i) no reasonable conclusions can be made if only based on UCP2/UCP3 gene expression, because both proteins have a strong translational regulation and short life-time (Pecqueur et al., 2001; Rousset et al., 2007; Rupprecht et al., 2012) and (ii) the expression of each uncoupling protein is confined to definite tissues or cells. That is, we and several other groups have previously demonstrated that UCP2, which mRNA was found nearly ubiquitously (Fleury et al., 1997; Alan et al., 2009), is present at protein level exclusively in highly proliferating cells, which have a glycolytic type of metabolism, such as cancer, stem and activated immune cells (Pecqueur et al., 2001; Alves-Guerra et al., 2003; Zhang et al., 2011; Rupprecht et al., 2012, 2014; Yu et al., 2013). In contrast, the expression pattern of UCP3, which was only found in brown adipose tissue, heart and SkMs, may imply a connection to another type of metabolism – fatty acid β-oxidation (FAO) (Hilse et al., 2016) - the hypothesis, which we have tested in the present study.

For this, we compared cell oxygen consumption, abundance of UCP2/UCP3, key mitochondrial proteins (VDAC, respiratory chain proteins), specific cardiomyocyte markers (SERCA2, GATA4 and troponin), specific markers of FAO and mitochondrial morphology during (1) murine (heart) development and (2) stem cell differentiation (mESC) to CM. The main advantage of these two models is that the behavior of both proteins can be studied during physiological alteration of the metabolic environment.

## Materials and Methods

### Animals and Tissue Samples Preparation

Embryonal, postnatal and adult females of C57BL/6 wt mice were used. The animals were kept in a 12:12 h light-dark cycle at room temperature and had unlimited access to food and water. Mice were sacrificed by decapitation or cervical dislocation in accordance with the guidelines for animal care and approved by the Ethical Committee of the University of Veterinary Medicine, Vienna. Tissues were shock-frozen in liquid nitrogen or fixed in 3% glutaraldehyde for electron microscopy immediately after preparation. Murine hearts were homogenized in RIPA buffer supplemented with a protease inhibitor cocktail using a mixer mill (MM200, Retsch, Germany) and then sonicated (Hilse et al., 2016). After 30 min of incubation on ice, the lysates were centrifuged 2 × 10 min at 2500*g*. The supernatants were collected, aliquoted and stored frozen at -80°C. The total protein concentration was determined with a Pierce BCA Protein Assay Kit (Thermo Fischer Scientific).

### Western Blot Analysis

Western blot analysis was performed as described previously (Hilse et al., 2016). We used antibodies against UCP3 and UCP2 [evaluated in (Hilse et al., 2016) and (Rupprecht et al., 2012), respectively], CII (SDHA, succinate dehydrogenase complex subunit A, Abcam ab14715, dilution 1:7500), GAPDH (glyceraldehyde-3-phosphate-dehydrogenase, Sigma-Aldrich G8795 dilution 1:7500), CI (NDUFA9, Invitrogen 459100, dilution 1:3000), CV (ATP-Synthase, β–subunit, Invitrogen A-2135, dilution 1:5000), α-actin (Abcam ab88226, dilution 1:3000), GATA4 (Santa Cruz sc-25310, dilution 1:1500), TnC (Troponin T-C, Santa Cruz sc-20025, dilution 1:1500), TnI (Troponin I, Santa Cruz sc-365446, dilution 1:1500), Serca1 (sarco-/endoplasmic reticulum calcium-ATPase 1, Santa Cruz sc-515162, dilution 1:1500), Serca2 (sarco-/endoplasmic reticulum calcium-ATPase 2, Abcam ab2861, dilution 1:7500), VDAC (Abcam ab14734, dilution 1:5000) and Oct 3/4 (Santa Cruz sc-9081; 1:1500). All antibodies were diluted in 2% BSA solution. Detection was performed by luminescence reaction using a secondary antibody against rabbit or mouse antibodies linked with horseradish peroxidase (GE Healthcare, Austria) and ECL Western Blotting reagent (Bio-Rad, Austria) using ChemiDoc-It 600 Imaging System (UVP, United Kingdom). The control proteins were detected on membranes prior to be stripped with a strip solution (100 mM sodium citrate at pH 2.2) for at least 1 min, washed and blocked for 30 min in block solution and incubated again with next control antibody.

We employed the Launch Vision Works LS software (UVP, United Kingdom) for quantification. The relative amount was calculated as a ratio between the sample and loaded standard (heart standard or mouse recombinant protein UCP2 (Hilse et al., 2016), see figure description). The heart standard was prepared from pooled hearts of 2–5 months old C57BL/6 wt mice (*n* = 15). 20 μg of the heart standard was loaded per lane.

### Cardiomyocyte Differentiation

We differentiated mESC (clone D3-ATCC) to CM according to the published protocol (Seiler and Spielmann, 2011). Stem cells were cultured in DMEM medium (high glucose with L-glutamine, without sodium pyruvate; Thermo Fisher Scientific) supplemented with 15% fetal calf serum, 2 mM glutamine, 1% non-essential amino acids, antibiotics (penicillin/ streptomycin solution 50 U/ml) and 0.1 mM β-Mercaptoethanol. The maintenance medium was supplemented with mLIF (murine leukemia inhibitory factor, 10^6^ U ml^-1^; Millipore), which was added directly to the plate for maintaining pluripotency and to avoid differentiation. Cells were passaged every 2–3 days. Differentiation into CM was initiated after 2 days in culture as a hanging drop after LIF omission. Formed EB were splashed into petri dishes and cultured for the subsequent 2 days. Finally, EB were transferred into 6-well plates and cultured for a maximum of 28 days in humidified atmosphere under normoxic conditions (5% CO_2_ and atmospheric oxygen 21% O_2_) at 37°C with a change in half the amount of medium every 3–4 days.

### Confocal Microscopy

To visualize the mitochondria, mESC and mESC-derived CM (at day 14) were incubated with 12.5 nM TMRE for 20 min and measured with an inverse confocal laser scanning microscope (Leica TCS SP5 II) as described in Zimmermann et al. (2017). Cells were kept under the microscope in a heating box (37°C) supplied with 5% CO_2_. TMRE was excited with a DPPS laser at a wavelength of 561 nm. Fluorescence was collected through a 40× oil immersion objective in an emission channel of 570 – 690 nm (512 × 512 pixels; scan speed 200 Hz). Images were recorded at intervals of 133 ms (256 × 256 pixel; scan speed 1000 Hz) for determination of the cell beat frequencies.

### Electron Microscopy

Samples (cell cultures and mouse hearts) were fixed in 3% buffered glutaraldehyde (pH 7.4, Merck, Darmstadt, Germany). The cell culture pellets were pre-embedded in 1.5% agar and washed with 0.1 M Sorensen phosphate buffer at pH 7.4. All samples were subsequently fixed in 1% osmium tetroxide (Electron Microscopy Sciences, Hatfield, United States) followed by dehydration in a series of ethanol dilutions (70, 80, 96, and 100%), embedded in epon-resin and polymerised 48 h at 60°C. Semi-thin sections (0.8 μm) were stained with toluidine blue, ultra-thin sections (70 nm) were mounted on copper grids (Gröpl, Tulln, Austria) and stained with uranyl acetate and lead citrate. Transmission electron micrographs were made with EM900 (Zeiss, Oberkochen, Germany).

### Determination of the Oxygen Consumption Rate (OCR) and Extracellular Acidification Rate (ECAR)

Mouse embryonic stem cell cells were seeded on gelatine-coated Seahorse 96XFe plates (Agilent) at least 16 h before analysis with an amount of 30,000 cells per well in the maintenance media (80 μl/well). To analyze differentiated mESC at day 14 (d14), one at d5 formed EB were seeded per well on gelatine-coated Seahorse 96XFe plates and grown for 9 days in maintenance media. Half of the media was changed every 2–3 days.

For determination of the energetic profile, medium was changed to 180 μl/well unbuffered XF base media (pH 7.4) an hour prior to analysis. XF base media contained 20 mM glucose, 5 mM glutamine and 1xNEEA equivalent to the content of the maintenance media. The cells were two times washed and then incubated with the XF base media in a non-CO_2_ incubator.

Oxygen consumption rate and ECAR were determined in parallel using the Seahorse XFe96 extracellular flux analyzer (Agilent) according to standard protocol, but for a measuring time of 2 min. After the experiment, cells were washed with PBS and 20 μl RIPA per well was added. The plate was stored at -20°C before protein determination by Pierce BCA Protein Assay Kit. Each experiment was performed on a new plate using independent subcultures of mESC and differentiation experiments. Only the inner 60 wells were taken into account. OCR and ECAR were normalized to μg protein per well and calculated as a mean with standard derivation from at least 14 technical replicates per energetic profile experiment.

### RNA Isolation and Quantitative Real Time PCR

Shock frozen hearts from E18, P1 and P7 and P30 were homogenized in liquid nitrogen using a mixer mill (MM200, Retsch, Germany). RNA from homogenized tissue and cells (mESC, d10, d19) samples were extracted in TRI Reagent (Invitrogen) according to manufacturer’s protocol. RNA concentration was determined using NanoDrop^TM^ (Thermo Fisher Scientific). One microgram of isolated RNA was used for cDNA production with the “High Capacity cDNA reverse Transcription kit” (Applied Biosystems). Quantitative real time PCR was performed with the QuantiFast Multiplex PCR +R Kit (Qiagen) and specific probes of TaqMan^TM^ Gene Expression Assay for following genes: Slc27a6 – Mm01258609_m1, Cpt1b – Mm00487191_g1, Acadvl – Mm00444293_m1, Ppargc1a - Mm01208835_m1 ID 4453320 and GAPDH – Mm99999915_g1 ID 4448484. The qRT-PCR was run on StepOnePlus (Applied Biosystem) and the software StepOne (Applied Biosystem) was used for data analysis. Melting curve calculations were done for all assays. Target genes were normalized to GAPDH as a housekeeping gene.

### Statistical Analysis

Data from WB analysis are presented as a mean ± SEM from at least three independent experiments. Data were analyzed with a *t*-test or one-way ANOVA by more than two groups. Data were considered to be statistically significant at ^∗^*p* < 0.05, ^∗∗^*p* < 0.01 and ^∗∗∗^*p* < 0.001 The analysis was carried out using Sigma Plot 12.5 software.

## Results

### Expression of Highly Homologous UCP3 and UCP2 in Heart Is Different During Embryonic Stage and Development of Mice

**FIGURE 1:**
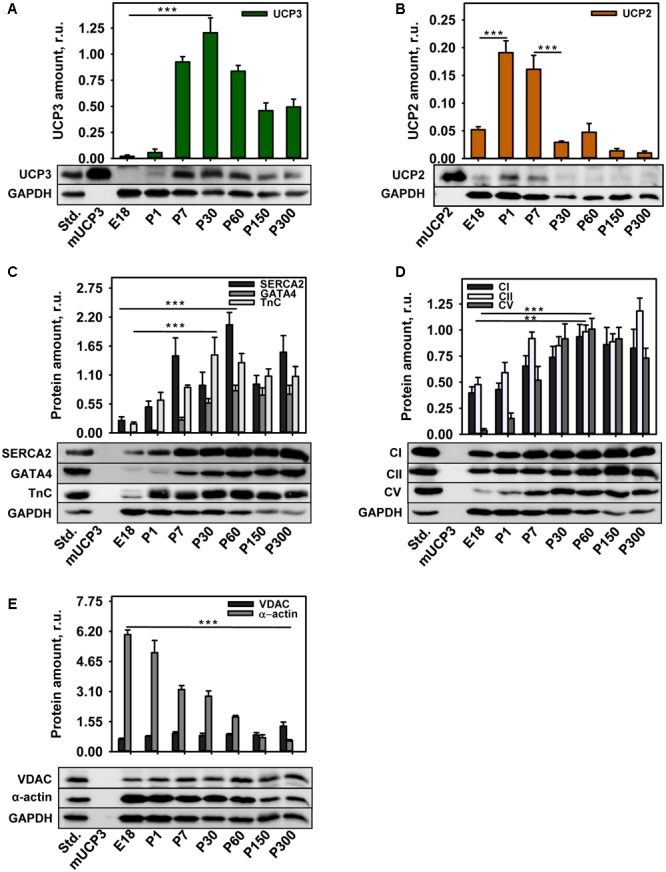
UCP3 and UCP2 expression during heart development. **(A–E)** Representative WBs and quantification for UCP3 **(A)** and UCP2 **(B)**, cardiac proteins – SERCA2, GATA4, Troponin C **(C)**, respiratory chain proteins of the inner mitochondrial membrane Complex I, II, V **(D)**, outer mitochondrial membrane protein VDAC, and cardiac muscle marker α-actin **(E)** in mice during heart development. The relative protein amounts were calculated as a ratio between line intensity of sample to standard (heart standard or mUCP2). 20 μg of total protein from tissue were loaded per lane. Recombinant mouse UCP3 (mUCP3, 5 ng) and mUCP2 (2.5 ng) were used as positive controls. GAPDH was used as a loading control. At least 5 samples of pooled embryos (E18) or postnatal day 1-300 (P1-P300) heart tissue were analyzed. Data are presented as mean values ± SEM, ^∗∗^*p* < 0.01 and ^∗∗∗^*p* < 0.001.

To address the conflicting reports about the presence of both proteins - UCP2 and UCP3 - in heart we analyzed UCP2 and UCP3 content at different time points of heart development: at embryonic stage (E18), one and 7 days after birth (P1 and P7), at two growth stages (P30 and P60) and in adult mice (P150 and P300) using antibodies that we have previously evaluated (Hilse et al., 2016). **Figure 1A** shows that UCP3 is not expressed in embryonic heart (E18) and first appears after birth with an expression peak at day 30, followed by a slight decline during the aging process. In contrast, we detected UCP2 already in embryonic heart, it has its expression peak in the 1st week after birth and vanishes after 1 month at the latest (**Figure 1B**; Supplementary Figure S1).

In the next step we evaluated whether UCP3 and UCP2 abundance correlated with the presence of proteins, which serve as markers for differentiated adult CM. **Figure 1C** shows that the expression of SERCA2 (Ca^2+^-ATPase type 2, specifically localized in sarco/endoplasmic reticulum), GATA4 (transcription factor expressed during myocardial development and growth) and TnC (subunit of the troponin complex, which is responsible for muscle contraction) started at E18 and achieved its maximum between 30 and 60 days, largely coinciding with the expression pattern of UCP3 (**Figure 1A**). To evaluate whether there is a putative connection between UCP3 and the mitochondrial RC function, we measured the abundance of CI, CII, and ATP synthase (CV) at different developmental stages. **Figure 1D** demonstrates that CI and CII are already present in embryonic heart in high amounts. All three proteins achieved the expression maximum at P60. Whereas CI and CV declined slightly with aging, CII abundance increased steadily up to 10 months after birth. In contrast, the expression of the VDAC, known to transport metabolites through the outer mitochondrial membrane (Colombini, 1979; Noskov et al., 2016), was nearly constant in embryonic heart and over the first 5 months of mouse life (**Figure 1E**). Notably, α-actin – a commonly used structural marker for skeletal and heart muscles – was highly abundant at E18 and showed a continuous reduction in amount with an increase in age (**Figure 1E**).

### UCP3 Is Not Involved in Electrical Activity and Contractility of Cardiomyocytes

**FIGURE 2:**
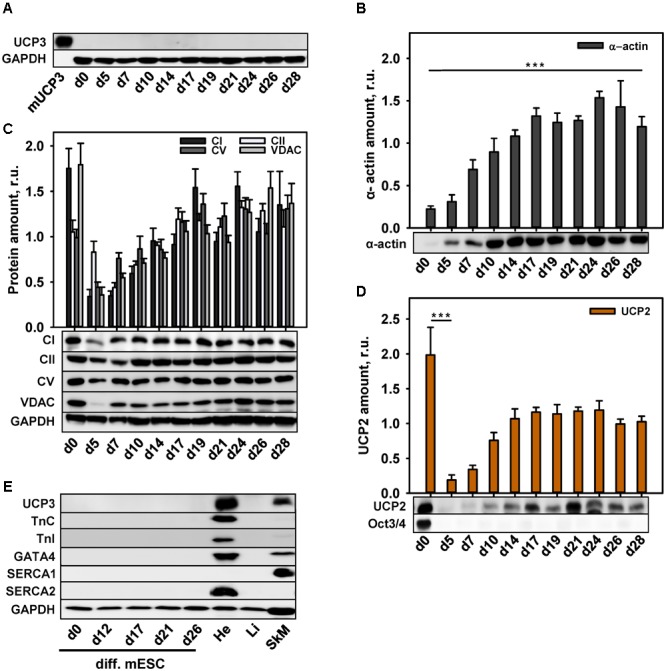
Protein expression during cardiomyocytes differentiation. **(A–E)** Representative WBs and quantification analysis of UCP3 **(A)**, α-actin **(B)**, mitochondrial markers – CI, CII, CV, VDAC **(C)**, UCP2 **(D)** and cardiac markers Troponin C, Troponin I, GATA4, SERCA2 and muscle marker SERCA1 **(E)** during cardiomyocytes differentiation. Antibody against OCT3/4 was used as a stem cell marker; Antibody against GAPDH was used as a loading control. Recombinant mouse UCP3 (mUCP3, 5 ng) was used as a positive control. 20 μg of total protein from cell lysate were loaded per lane. Cells were collected at different time points from 3 to 6 independent differentiation experiments. The relative protein amounts were calculated as a ratio between the line intensity of sample to the intensity mean of all values loaded on the membrane. Data are presented as mean values ± SEM, ^∗∗∗^*p* < 0.001.

To correlate UCP3 expression with the degree of cell maturity, we differentiated murine embryonic stem cells (mESC) to CM according to the protocol described previously (Seiler and Spielmann, 2011). In agreement with the protocol, we routinely observed cell contractions with frequency 1–2 Hz after 12 days of cell differentiation (Supplementary Video 1). Surprisingly, we failed to detect UCP3 at any stage of mESC differentiation (**Figure 2A**), although mESC-derived CM showed the expression of α-actin (**Figure 2B**).

To control whether the abundance of other mitochondrial proteins was impaired at any point of mESC differentiation, we traced the expression behavior of VDAC, CI, CII, CV, and UCP2. **Figure 2C** reveals that stem cells displayed a high expression of RC proteins indicating their high metabolic activity. The expression was declined in EB (d5) and increased again after day 7 in culture, basically mirroring the expression pattern of RC proteins during murine heart development (**Figures 1D,E**). UCP2 was abundant not only in stem cells, but also at the late stages of differentiation (**Figure 2D**), implying that the mESC, differentiated in this way, still have features of stem cells despite the absence of Oct3/4. To support this hypothesis, we tested the expression of the specific CM markers. **Figure 2E** demonstrates that TnC, Troponin I, GATA4 and SERCA2 were not present during the whole differentiation time. The absence of Ca^2+^-ATPase type 1 (SERCA1) specifically expressed in muscles (**Figure 2E**), allowed us to exclude the differentiation of stem cells in myoblasts.

### Appearance of Lipid Drops Coincides With High Expression of UCP3

**FIGURE 3:**
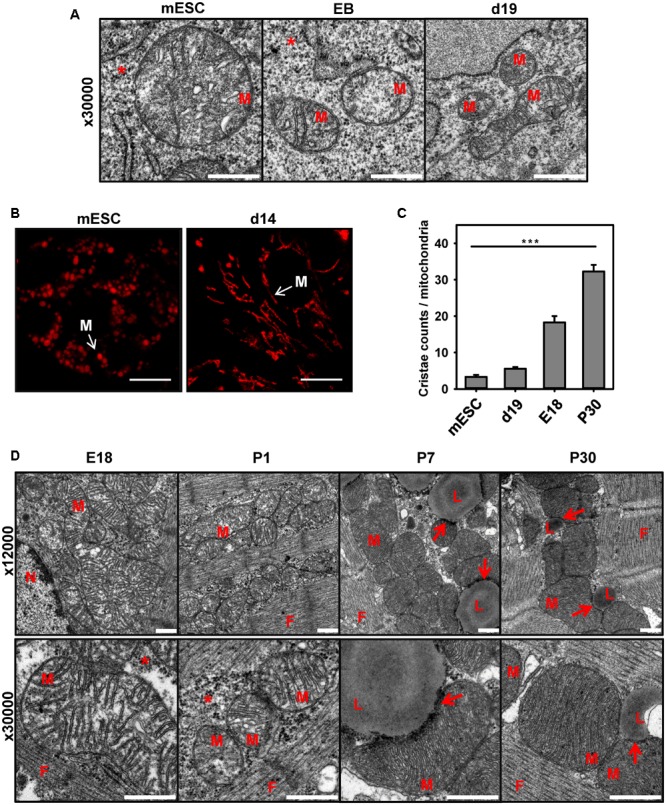
Comparison of mitochondria shape and cristae during mESC differentiation to cardiomyocytes and heart development. **(A)** Representative electron microscopy pictures of mouse embryonic stem cells (mESC, left) embryo bodies (EB, middle) and mESC-derived CM at day 19 (d19, right). Detailed images of mitochondria at magnification 30000X are shown. Scale bar – 500 nm. **(B)** Representative TMRE fluorescence images of mESC and mESC-derived CM at d14. Scale bar - 10 μm. Pictures were taken from 2 to 3 independent differentiation experiments. **(C)** Quantification of cristae amount per mitochondria. Data are presented as mean values ± SEM, ^∗∗∗^*p* < 0.001. **(D)** Representative electron microscopy pictures of mitochondria heart tissue (magnification 12000× top, and 30000× bottom) in E18, newborn day 1 (P1), day 7 (P7) and adult day 30 (P30) mice. M, mitochondria; N, nucleus; L, lipid droplets; F, cardiac myofibrils; arrow - LD/Mitochondria contact; ^∗^ - glycogen. Embryos (E18) or postnatal (P1-30) heart tissue from at least two different animals were analyzed per group. Scale bar – 500 nm.

Analysis of the expression pattern of cardiomyocyte markers presented in **Figure 2E** led us to the hypothesis that the cell’s mitochondria in the model of stem cell differentiation are still immature, despite the existing electrical activity and presence of α-actin. To evaluate this, we visualized the mitochondrial shape and number of cristae at three time points during stem cell differentiation using electron microscopy. **Figure 3A** demonstrates that mESCs have mostly round mitochondria with a low cristae density. At the start of differentiation, the size of mitochondria in EB decreased and the density of cristae remained low (**Figures 3A,C**). At day 14 mitochondria exhibited more variability showing round and elongated shapes (**Figure 3B**), probably indicating the higher fusion/fission rate (Chen and Chan, 2005; Berman et al., 2008; Benard and Karbowski, 2009), but still had a lower number of cristae, when compared to CM from adult heart (**Figures 3C,D**).

The electron microscopy of CM from postnatal mice (P7 and P30) revealed the appearance of big lipid droplets (**Figure 3D**; L), which were absent in differentiated stem cells (**Figure 3A**) and embryonic cells, having large stores of glycogen instead (**Figure 3D**). LDs are known as storage organelles for triglycerides, which are used by mitochondria in the β-oxidation pathway (FAO) (Farese and Walther, 2009; Schulze et al., 2016; Schuldiner and Bohnert, 2017). We detected direct contacts that several mitochondria of mature CM built to the LD surface (**Figure 3D**, arrows). This type of contact between mitochondria and lipid droplets was previously described for animals under high-fat diets or diabetes type 2 (Aon et al., 2014).

### Expression of UCP2 and UCP3 Depends on Type of Cell Metabolism

**FIGURE 4:**
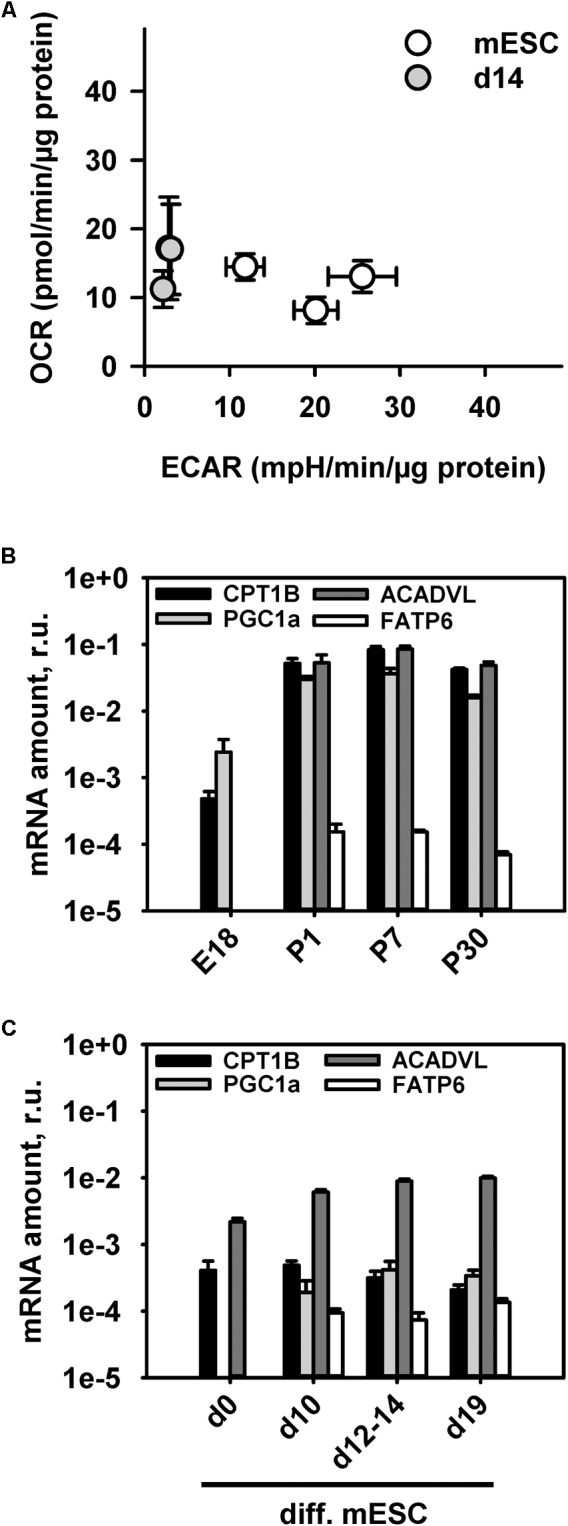
Characterization of cell metabolism during stem cell differentiation into cardiomyocytes using the extracellular flux analyzer. **(A)** Determination of the energetic profile of mESC (open circles) and mESC-derived CM at d14 (gray filled circles) using extracellular flux analyzer under standard media conditions from three independent experiments. **(B,C)**. Quantitative RT-PCR mRNA transcript analysis of β-oxidation markers (PGC1a, FATP6, CPT1B, and ACADVL) at different time points during mouse heart development **(B)** and mESC differentiation into cardiomyocytes **(C)**. Results are presented relative to mRNA amounts of the housekeeping gene GAPDH. Each data point represents the mean value ± SEM of at least *n* = 3 animals per age.

It is well-known that adult CM predominantly have a FAO type of metabolism (Lopaschuk and Jaswal, 2010). To support the idea that immature mitochondria in mESC-derived CM have a different metabolism than adult CM, we performed measurements of the OCR and ECAR in mESC-derived CM at day 14 using Seahorse extracellular flux analyzer (**Figure 4A**). Analysis of the OCR and ECAR in media containing the same amounts of glucose and amino acids as the standard “maintenance media” revealed a glycolytic type of energy metabolism similar to that of the undifferentiated mESC with a slight shift to an oxidative metabolism (**Figure 4A**). The more quiescent metabolism of mESC-derived CM at d14 reflects less energetic requirements than in adult CM, and shows that they are not fully active, in comparison to the highly proliferative mESC.

To establish another link between UCP3 expression and FAO in developing heart and CM under differentiation we evaluated mRNA transcripts of several proteins which were demonstrated to be important at different steps of the β-oxidation pathway such as transcriptional co-activation and mitochondrial biogenesis (PGC1a), cellular FA transport (FATP6/SLC27A6), mitochondrial FA uptake (CPT1B) and FA enzymatic cleavage (ACADVL) (Huss and Kelly, 2004; Black et al., 2009; Lopaschuk and Jaswal, 2010; Rowe et al., 2010). To increase marker specificity for heart cardiomyocyte-abundant isomers were chosen. We observed a rise of PGC-1α expression from embryonic to postnatal stage (**Figure 4B**). This confirms a known sharp PGC-1α expression increase directly after birth which was shown to coincide with a perinatal shift from glucose metabolism to FAO (Huss and Kelly, 2004). mESC-derived CMs showed only slight PGC-1α increase during their differentiation (**Figure 4C**), which not reached the same expression level as in young hearts or even embryonic heart (**Figure 4B**). Similarly, the mitochondria-associated CPT1B and ACADVL showed a rise in their transcript levels during heart development, but a constantly low expression level during CM differentiation. Interestingly, the heart specific FATP6, which is responsible for the cellular FA uptake showed the same expression pattern during heart development and cardiomyocyte differentiation. PGC1 and FATP6 gene expression was below the detection limit in stem cells and in E18 hearts.

## Discussion

By using mESC-derived CM in this study, we have revealed that the appearance of UCP3 in heart cannot be attributed to the establishment of electrical/contractile activity in CM, which is the main criterion for the successful mESC differentiation in all protocols (Seiler and Spielmann, 2011; Niehoff et al., 2016). UCP3′s presence is rather an indicator of CM’ maturity, which includes (i) the presence of specific CM markers – SERCA2, TnC and GATA4 (**Figure 1C**), (ii) an increase of cristae density in mitochondria (**Figure 3C**), (iii) the appearance of lipid droplets (LD) and the establishment of multiple contacts between LD and mitochondria (**Figure 3D**, arrows), and, the most important, (iv) a switch to FAO as a main type of cell metabolism (**Figure 4** and (Lopaschuk and Jaswal, 2010; Mdaki et al., 2016)). These findings are in agreement with the time course of UCP3 expression during heart development (**Figure 1A**) and with several studies implying the correlation between UCP3 and metabolism (Hilse et al., 2016). It was shown at gene expression level that UCP3 was downregulated in fetal and failing human heart (Razeghi et al., 2001) and was increased by ligand activation of PPARα (Young et al., 2001), known as a major regulator of genes involved in the lipid metabolism in liver, heart, and muscle (Warren et al., 2017).

Whereas UCP3 revealed a strong time correlation with proteins indicating the differentiation stage of cells and the maturity of the metabolism, we observe no time coincidence between UCP3 and RC proteins abundance (RCP, **Figure 1D**), implying a weak connection between the function of these proteins. Moreover, the decrease in UCP3 abundance found in mitochondria of aging heart is in line with the impairment in FAO and a shift toward carbohydrate metabolism (Lesnefsky et al., 2016). Therefore, UCP3 expression can reflect the FAO capacity and indicate metabolic impairments in adult heart.

UCP3 is supposed to be localized on the inner boundary of the mitochondrial membrane, analogous to other uncoupling proteins (Klotzsch et al., 2015), and was shown to transport protons with a similar turnover rate as UCP1 and UCP2 (Urbankova et al., 2003; Beck et al., 2007; Macher et al., 2018). However, other transported molecules, such as FA (Schrauwen et al., 2001), fatty acid peroxides (Goglia and Skulachev, 2003; Lombardi et al., 2010; Senese et al., 2011) and pyruvate (Criscuolo et al., 2006) instead or alongside with protons, were also proposed. This additional transport pathway is in line with the putative involvement of UCP3 in (fatty acid) metabolism, which has been described for muscles (Clapham et al., 2000; Himms-Hagen and Harper, 2001) and for adult CM in this work. Our present results which reveal the tight correlation between increase in UCP3 amount, expression of mitochondrial FAO maturation markers and establishment of multiple connections between mitochondria and LD may support the hypothesis that UCP3 facilitates FAO metabolism by transporting FAs. Although the transport of FA outside mitochondria as a function of UCP3 was already proposed by several groups (Himms-Hagen and Harper, 2001; Schrauwen et al., 2006), the tight connection between huge lipid droplets and mitochondria at the peak of UCP3 expression let us speculate that UCP3 may transport FAs inside mitochondria.

In contrast to the reports of several research groups (Cabrera et al., 2012; Perrino et al., 2013), we have not found UCP2 in adult murine heart under physiological conditions. However, we detected UCP2 in mESC and in embryonal/neonatal heart (**Figure 1B**) alongside with low cristae density and increased glycogen stores (**Figure 3D**) as typical features of tissues with glycolytic type of metabolism (Lopaschuk et al., 1991). It is known that mammalian CM can avidly proliferate during fetal and neonatal development (Xin et al., 2013; Foglia and Poss, 2016). The rapid ventricular remodeling that occurs over the few days after birth can explain the additional UCP2 expression peak, which we detected between P1 and P7. We also revealed UCP2 in mESC-derived CM alongside with glycolytic type of metabolism observed in extracellular flux analysis experiments (**Figure 4**). These results additionally support the idea that CM’s contractile activity and the expression of the alpha-actin are not sufficient to regard these cells as mature CM. It was reported earlier that UCP2 modulates myocardial excitation-contraction coupling (Turner et al., 2010) and/or attenuates ROS generation (Teshima et al., 2003) if ectopically expressed in neonatal CM.

Previously, we have observed that another uncoupling protein, UCP4, can be detected in brain and in neurons derived from the same mESC, but only simultaneously with the appearance of neuronal cell markers which indicated the switch from glycolytic metabolism to oxidative phosphorylation (Smorodchenko et al., 2009, 2011; Rupprecht et al., 2014). Notably, the neuroblastoma cell line, usually used as a model for primary neurons, expresses UCP2 instead of UCP4 (Rupprecht et al., 2014) coinciding with the expression of cancer cell markers and glycolytic cell metabolism. The expression pattern of uncoupling proteins, therefore, strengthens the new concept about their involvement in metabolism in stem cells and immature CM on the one side (UCP2), and adult CM on the other side (UCP3).

## Conclusion

**FIGURE 5:**
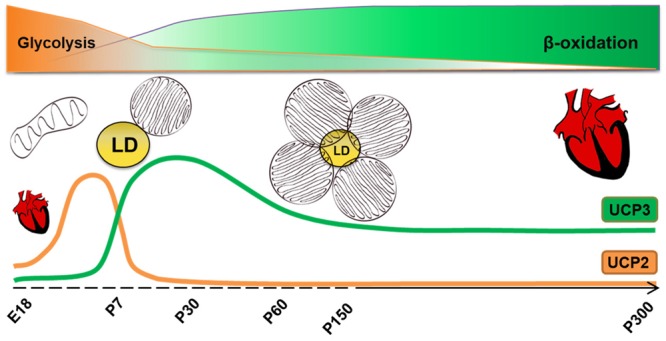
Correlation between metabolism type, morphology of mitochondria and UCP2/UCP3 protein content in cardiomyocytes at different time point of heart development. LD, lipid droplets, E18-embryonic day 18.

We have demonstrated a clear overlap between a cellular bioenergetics profile, lipid droplet accumulation, expression of specific CM maturity and FAO markers and UCP3 protein content in heart tissue at different time point of the development (**Figure 5**). Understanding that the expression patterns of UCP3 and UCP2 in heart are different and reflect the metabolic type and cell differentiation degree is an important step for uncovering their exact functions in CM metabolism.

## Author Contributions

EP and KH contributed to the conceptualization. KH, AR, AS, ME, and LZ contributed to the methodology. KH, AR, SB, and LZ contributed to the investigation. EP, AS, and ME provided the resources. KH and EP wrote the original draft. KH, AR, and EP reviewed and edited the draft. KH, AR, LZ, and EP contributed to the visualization. EP acquired funding and supervised the work.

## Conflict of Interest Statement

The authors declare that the research was conducted in the absence of any commercial or financial relationships that could be construed as a potential conflict of interest.

## Abbreviations

CI: complex I
CII: succinate dehydrogenase complex subunit A
CM: cardiomyocytes
CV: ATP-Synthase, β–subunit
d14: day 14 of differentiation
E18: embryo day 18
EB: embryo bodies
ECAR: extracellular acidification rate
FA: fatty acids
FAO: fatty acid oxidation
GAPDH: glyceraldehyde 3-phosphate dehydrogenase
He: heart
Li: liver
mESC: mouse embryonic stem cells
mLIF: murine leukaemia inhibitory factor
mUCP3: mouse recombinant UCP3
OCR: oxygen consumption rate
P1: postnatal day 1
RC: respiratory chain
ROS: reactive oxygen species
Serca2: sarco/endoplasmic reticulum calcium-ATPase 2
SkM: skeletal muscle
Std: standard
TnC: Troponin C
TnI: Troponin I
TMRE: tetramethyl rhodamine ethyl ester
UCP3: uncoupling protein 3
VDAC: voltage-dependent anion channel
WB: Western blot
wt: wild type mouse
