# Supplementary material for: The Expression of Uncoupling Protein 3 Coincides With the Fatty Acid Oxidation Type of Metabolism in Adult Murine Heart
Source: Front Physiol. 2018 Jun 22;9:747. doi: 10.3389/fphys.2018.00747 (PMC6024016; doi:10.3389/fphys.2018.00747)
Supplement: Supplementary file 2 [file Presentation_1.PDF]

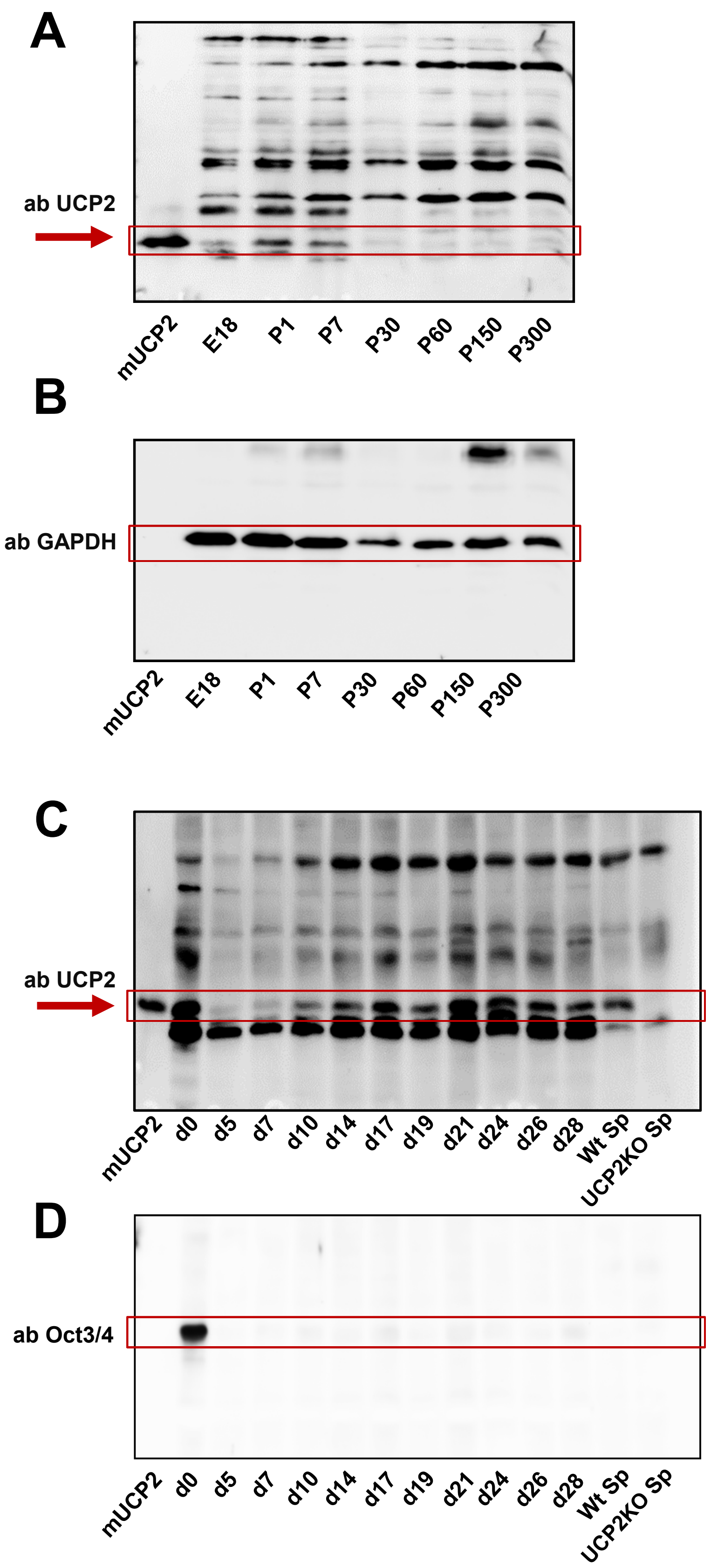

Figure S1. Whole WB images showing the expression of UCP2 (A) and GAPDH (B) in murine hearts at different postnatal days and UCP2 (C) and Oct3/4 (D) in cardiomyocytes derived from mESC at different differentiation days. Antibody against UCP2 was validated with recombinant UCP2 (mUCP2) and spleen from UCP2 KO (negative control) and wt mice KO (positive control).
